# How much preoperative flexion contracture is a predictor for residual flexion contracture after total knee arthroplasty in hemophilic arthropathy and rheumatoid arthritis?

**DOI:** 10.1186/s43019-022-00146-2

**Published:** 2022-04-08

**Authors:** Hyun Woo Lee, Cheol Hee Park, Dae Kyung Bae, Sang Jun Song

**Affiliations:** 1 Department of Orthopaedic Surgery, College of Medicine, Kyung Hee University, 26 Kyunghee-daero, Dongdaemun-gu, Seoul, 130-702 Korea; 2 Department of Orthopaedic Surgery, Seoul Sacred Heart General Hospital, Seoul, Korea

**Keywords:** Knee, Hemophilia, Rheumatoid arthritis, Arthroplasty, Flexion contracture, Complications

## Abstract

**Background:**

Although total knee arthroplasty (TKA) in hemophilic arthropathy (HA) or rheumatoid arthritis (RA) can improve functional ability, the postoperative range of motion (ROM) and prosthesis durability are reduced compared with those in osteoarthritic patients.

**Aim:**

We aimed to compare (1) the pre- and postoperative flexion contracture after TKA in HA and RA, (2) the threshold of preoperative flexion contracture as a predictor of residual contracture > 15° after TKA, and (3) the survival rate.

**Methods:**

Data from a consecutive cohort comprising 48 TKAs in HA and 92 TKAs in RA were retrospectively reviewed. The degree of flexion contracture was analyzed. Through receiver operating characteristics analysis, we aimed to determine the cutoff value of preoperative flexion contracture that increases the risk of residual contracture > 15° after TKA and compare the cutoff value in HA and RA. The survival rate was evaluated based on life table analysis and the Kaplan–Meier method.

**Results:**

The degree of preoperative flexion contracture was not significantly different. The degree of postoperative residual flexion contracture was 5.6° in the HA group and 1.4° in the RA group, respectively (*p* < 0.001). The cutoff value of preoperative flexion contracture for residual contracture of > 15° at last-follow up was 25.0° in the HA group and 32.5° in the RA group. The 5- and 12-year survival rates were 96% and 87% in the HA and 99% and 95% in the RA group, respectively (n.s.).

**Conclusions:**

The postoperative residual flexion contracture was greater and the cutoff value of preoperative flexion contracture for residual contracture was smaller in the HA group than the RA group. Appropriate intra- and postoperative care to avoid postoperative residual contracture is required in HA patients.

**Level of evidence:**

III.

## Background

Most existing literature on the results of total knee arthroplasty (TKA) has focused on patients with degenerative osteoarthritis (OA) because it is the most common type of knee arthritis [1–4]. Contrary to OA, HA and RA have similar disease characteristics, including preceding synovial hypertrophy and subsequent cartilage destruction, periarticular soft tissue contracture, high incidence of flexion and valgus deformity of the knee, poor bone quality, involvement of multiple joints, and long duration of disease progression [5]. TKA in both HA and RA is technically demanding due to extensive arthrofibrosis and combined severe deformities [6, 7], with a high rate of complications [8, 9]. Although efforts are made to delay the timing of surgery, TKA is often performed earlier in relatively young HA or RA patients due to the long disease duration and functional disability.

Even slight loss of knee extension may have a significant negative impact on gait and functional ability. Flexion contracture increases energy consumption and places undue strain on the quadriceps [10, 11]. Although surgeons have attempted to avoid residual flexion contracture during TKA procedures, massive soft tissue release can increase the risk of postoperative instability, especially in RA with severe flexion contracture [12]. Hwang et al. [13] performed TKA in RA according to the rule of one-third correction of flexion contracture and conducted serial casting and physical therapy, obtaining full extension with two-thirds gradual correction of flexion contracture. Atilla et al. [14] found that the flexion contracture at final follow-up was related to the severity of the initial preoperative flexion contracture, with a threshold of 27.5° to avoid residual contracture > 15° after TKA in HA patients. To the best of the authors’ knowledge, no previous study has compared the threshold of preoperative flexion contracture as a predisposing factor of residual flexion contracture after TKA in HA and RA.

Most studies on TKA in HA and RA indicate that complications such as periprosthetic joint infection (PJI) and loosening are more frequent than in patients with degenerative osteoarthritis [3, 15–18]. Song et al. [15] reported a high incidence of PJI and periprosthetic fracture (3 and 4 of 131 knees, 2.3% and 3.1%) after TKA in HA. Lee et al. [19] reported high risk of complication and revision TKA in RA. Revision TKA was necessary in 19 (15.9%) knees at a mean of 12.2 (range 6–16) years postoperatively. To the best of the authors’ knowledge, no studies have compared the incidence of complications and survival rate after TKA in HA and RA.

This study aimed to compare (1) the pre- and postoperative flexion contracture after TKA in HA and RA, (2) the threshold of preoperative flexion contracture as a predictor of residual contracture > 15° after TKA, and (3) the incidence of complications and survival rate.

## Patients and Methods

### Patients

For HA, 52 TKAs were consecutively performed in 41 patients aged < 65 years from 2002 to 2018 at the tertiary medical center. Indications for TKA were end-stage HA of the knee with Arnold–Hilgartner stage IV and V [20], gait disturbance and functional impairment due to severe pain and limited range of motion (ROM), and unresponsive to conservative treatment. For RA, 100 consecutive TKAs (70 patients aged < 65 years) during the same operating period were included. Indication for TKA was RA with functional impairment of the knee unresponsive to conservative treatment.

Patients without minimal 2-year follow-up were excluded, and four TKAs (four patients) in HA and eight (seven patients) in RA were excluded. The study was approved by our institutional review board (KHMC 21-04-093).

Demographic data are presented in Table 1. There was no significant difference in terms of preoperative flexion contracture and follow-up periods between the HA and RA groups (Tables 1 and 2).

**Table 1:** Patient demographics

	Hemophilia	Rheumatoid arthritis	*p*-Value
Number of knees (patients)	48 (37)	92 (63)	
Hemophilia type A/B^a^	32/5		
Hemophilia severity (severe/moderate)^b^	4/33		
HIV (negative/positive)	37/0	63/0	
HBV (negative/positive)	14/23	33/30	0.159
HCV (negative/positive)	10/27	63/0	< 0.001
Factor inhibitor (negative/positive)	31/6		
Age (years)	46.7 ± 11.2	57.4 ± 10.1	< 0.001
Female/male	3/34	58/5	< 0.001
Body mass index (kg/m^2^)	22.4 ± 2.8	25.0 ± 4.0	0.011
Right/left	26/22	49/43	0.919
Normal patellar height/patella baja	34/14	87/5	< 0.001
Operating period	2002–2018	2003–2018	
CR/PS/CCK^c^	1/46/1	9/80/3	0.321
PFC/Triathlon/Lospa/NexGen/Persona/Attune^d^	12/24/9/0/2/1	56/1/1/13/12/9	< 0.001
Surgical approach (MPPA/rectus snip/V–Y quadricepsplasty)	44/1/3	92/0/0	0.013
Patella resurfacing/patella non-resurfacing	46/2	86/6	0.715
Follow-up period (years)	8.8 ± 4.9	8.0 ± 4.6	0.389

*MPPA* medial parapatellar approach with general method

^a^Type A/B, patient deficient of coagulation factor VIII/IX

^b^Severe/moderate, factor level of < 1%/1–5%

^c^CR/PS/CCK, the types of prostheses implanted, including those retaining and substituting of the posterior cruciate ligament and constrained condylar knee

^d^PFC/Triathlon/Lospa/NexGen/Persona/Attune, PFC (Press Fit Condylar, Depuy, Raynham, MA), Triathlon (Stryker, Mahwah, NJ), Lospa (Corentec, Seoul, Korea), NexGen (Zimmer, Warsaw, Indiana), Persona (Zimmer, Warsaw, Indiana), Attune (Depuy, Raynham, MA)

**Table 2:** Clinical results

		Hemophilia	Rheumatoid arthritis	*p*-Value
Number of knees (%)		48 (34.3)	92 (65.7)	
Knee score	Preoperative	33.5 ± 5.2	41.2 ± 4.1	< 0.001
	Last follow-up	76.9 ± 7.3	82.3 ± 4.2	< 0.001
	Change	43.4 ± 6.9	41.1 ± 5.9	0.043
Functional score	Preoperative	33.4 ± 5.5	40.5 ± 3.8	< 0.001
	Last follow-up	78.1 ± 4.9	82.0 ± 4.7	< 0.001
	Change	44.7 ± 6.2	41.4 ± 6.3	0.004
WOMAC score	Preoperative	67.6 ± 4.2	63.8 ± 5.9	< 0.001
	Last follow-up	20.7 ± 2.3	17.9 ± 2.1	< 0.001
	Change	−46.9 ± 4.1	−45.9 ± 6.1	0.325
Flexion contracture (°)	Preoperative	16.5 ± 12.5	13.1 ± 13.8	0.159
	Last follow-up	5.6 ± 6.5	1.4 ± 4.5	< 0.001
	Change	−10.9 ± 11.0	−11.8 ± 12.4	0.673
Maximum flexion (°)	Preoperative	68.1 ± 32.0	117.2 ± 23.8	< 0.001
	Last follow-up	79.8 ± 21.8	128.6 ± 13.2	< 0.001
	Change	11.7 ± 27.6	11.4 ± 23.1	0.958
Range of motion (°)	Preoperative	51.9 ± 34.3	103.4 ± 30.8	< 0.001
	Last follow-up	74.2 ± 23.3	127.3 ± 14.1	< 0.001
	Change	22.3 ± 31.3	23.9 ± 28.9	0.758

*WOMAC* Western Ontario and McMaster Universities Osteoarthritis Index

### Operative technique

HA patients received coagulation factor replacement therapy according to our institutional protocol as per the World Hemophilia Foundation guidelines [6, 21]. The protocol was modified depending on patient’s factor levels, pharmacokinetic parameters such as recovery and half-life, and surgical wound status. Patients with high-titer inhibitors were treated with activated recombinant factor VIIa. Antithrombotic chemical prophylaxis was not used in HA patients but rather aspirin was used in RA patients.

The identical surgical technique of TKA with modified measured resection was applied in HA and RA patients [15]. General anesthesia is preferable due to the risk of spinal bleeding in HA patients [22]. A midline skin incision and medial parapatellar approach was used in all RA patients and most HA patients (Table 1). For proper exposure, a rectus snip was required in one knee, and V–Y quadricepsplasty was performed in three knees of the HA group (Table 1); these procedures were selectively performed only when there was a severe restriction on knee flexion because of concern about postoperative extension lag. Tibial tuberosity osteotomy was not applied in HA and RA patients due to poor quality of the periarticular bone [23]. Contemporary prostheses were implanted (Table 1). We preferred to additionally resect the distal femoral bone at 2–4 mm in severe flexion contractures > 20° rather than performing posterior capsulotomy. All components were implanted using bone cement mixed with 1 g of cephalosporin. Careful hemostasis was performed after deflating the tourniquet. Patellar resurfacing was performed in most knees, except in two and six knees with extremely small and thin patella in HA and RA patients, respectively (Table 1). The Hemovac drain was removed 2 days postoperatively, and active knee joint exercise was initiated. After 3 days, patients were mobilized with crutches until 1 month postoperatively. In patients who underwent rectus snip or V–Y quadricepsplasty, the ROM was restricted with a brace in extension, and the active extension and straight leg-raising exercise were withheld for 10 days to avoid postoperative extension lag. HA patients were discharged and referred to the Korea Hemophilia Foundation (KHF) for rehabilitation at a median of 9 days postoperatively. RA patients were discharged to home or convalescent center at a median of 7 days postoperatively. HA and RA patients had sequential follow-up at 6 weeks, 3 months, 6 months, and 1 year after surgery and were followed up annually thereafter.

### Clinical evaluation

The Knee Society knee and function scores [24] and Western Ontario and McMaster Universities Arthritis Index (WOMAC) [25] were used to evaluate pain and function both preoperatively and at last follow-up (Table 2). Flexion contracture, maximum flexion, and ROM were measured using a long-arm goniometer, with the patient in supine position.

### Radiographic evaluation

Serial pre- and postoperative anteroposterior and lateral radiographs and orthoroentgenograms were used to assess limb alignment, component position, and patellar height. Measurements were made on these images using a picture archiving and communication system (PACS) (Infinitt Healthcare Co. Ltd., Seoul, Korea). The patellar height was measured pre- and postoperatively using the Insall–Salvati ratio (ISR) and Blackburne–Peel ratio (BPR). Patella baja was defined as a truly low-lying patella due to shortening of the patellar tendon, which is associated with ISR of < 0.8 and BPR of < 0.54. Pseudo-patella baja was defined as a relatively low-lying patella to joint line, which is associated with normal ISR and BPR of < 0.54 [26].

### Complications

Any complications and need for additional surgery following primary TKA were reviewed according to the Knee Society classification system [27]. Prosthetic loosening was defined as occurring in knees with progressive radiolucent line > 2 mm wide on any radiograph, visible fracture of the cement around the components, or changes in component position, including subsidence [28].

### Statistical analysis

Preoperative and last follow-up values were compared using the paired *t*-test. Continuous variables between HA and RA groups were compared using Student’s *t*-test. Discrete variables were compared using the chi squared test and Fisher’s exact test. Preoperative and last follow-up values of flexion contracture and maximum flexion were compared using correlation analysis in both HA and RA groups. Through receiver operating characteristics (ROC) analysis, we aimed to determine the minimum amount of preoperative flexion contracture that increases the risk of residual contracture > 15° after TKA [14] and compare the cutoff value in HA and RA. Residual flexion contracture > 15° was thought to be clinically significant because it affects the patients’ walking ability [29].

The survival rate was evaluated based on life table analysis and the Kaplan–Meier method. The follow-up interval unit was 1 year, and annual success was defined as instances in which the implant remained in place throughout the time period.

All statistical analyses were performed using SPSS software (version 20.0; SPSS, Inc., Chicago, IL). In all analyses, *p* < 0.05 was considered statistically significant.

## Results

### Clinical evaluation

**Fig. 1:**
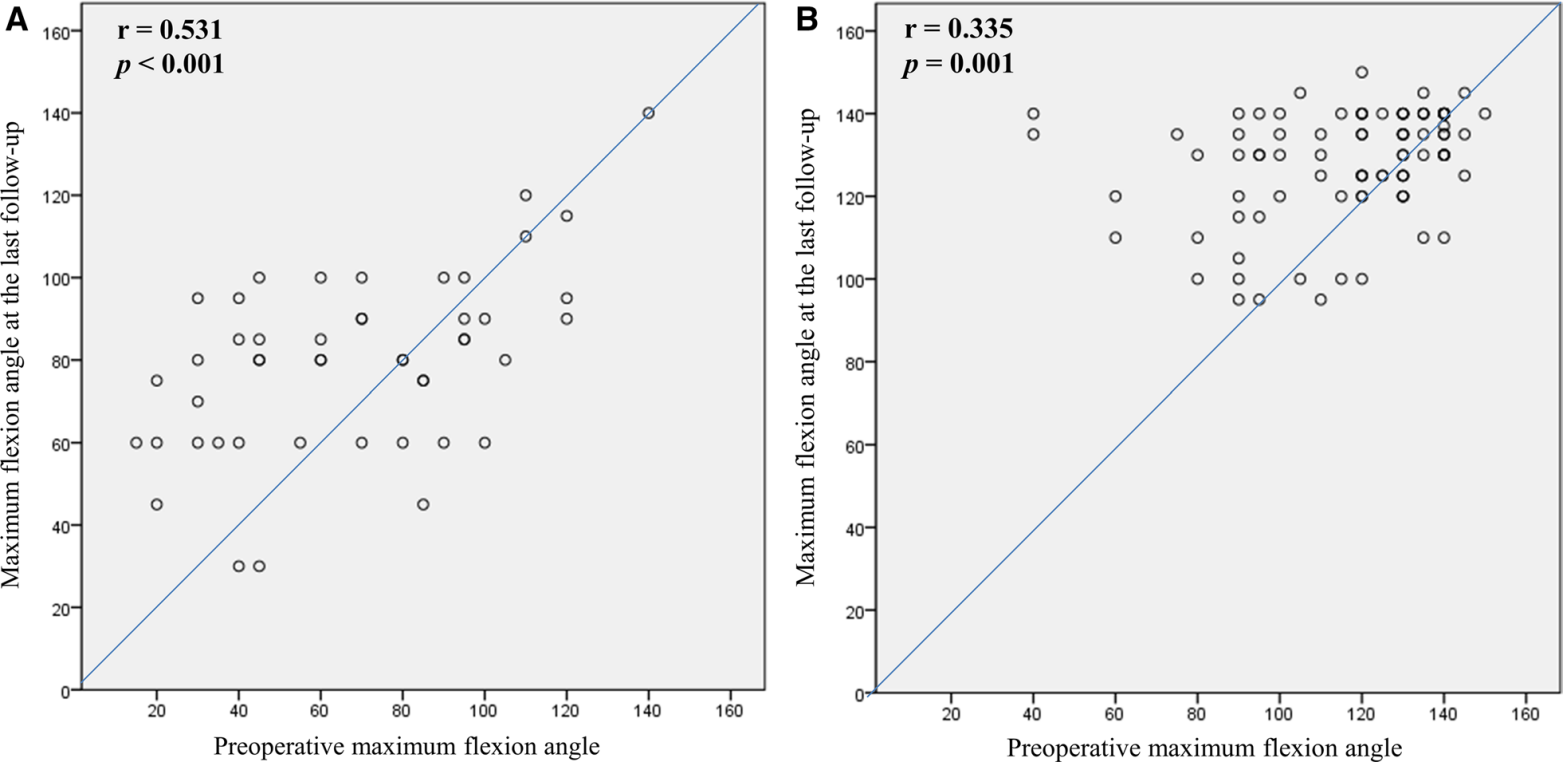
Correlation analysis between preoperative and last follow-up maximum flexion angles. **A** Hemophilic arthropathy. **B** Rheumatoid arthritis

**Fig. 2:**
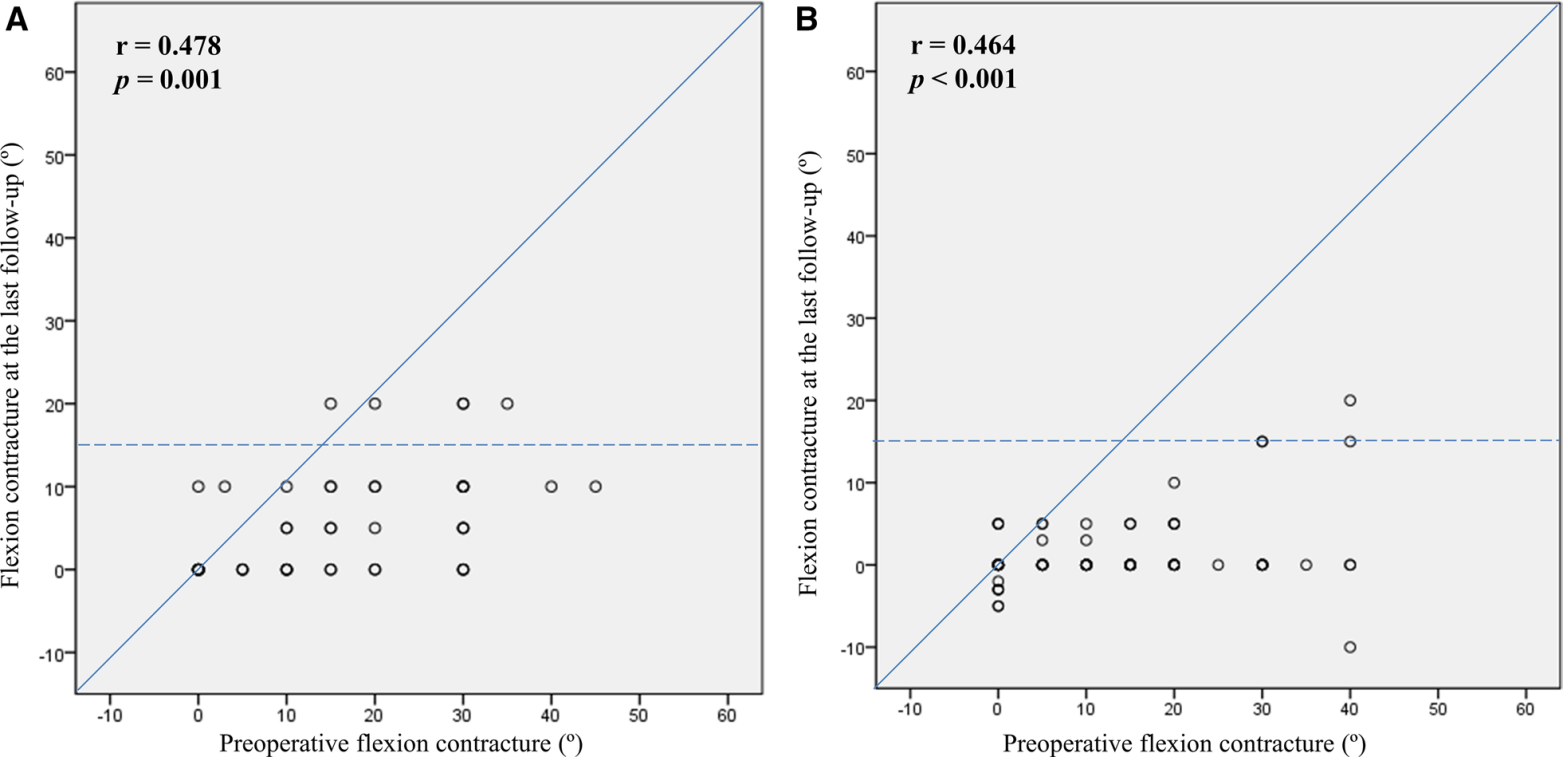
Correlation analysis between preoperative and last follow-up flexion contracture. **A** Hemophilic arthropathy. **B** Rheumatoid arthritis

**Fig. 3:**
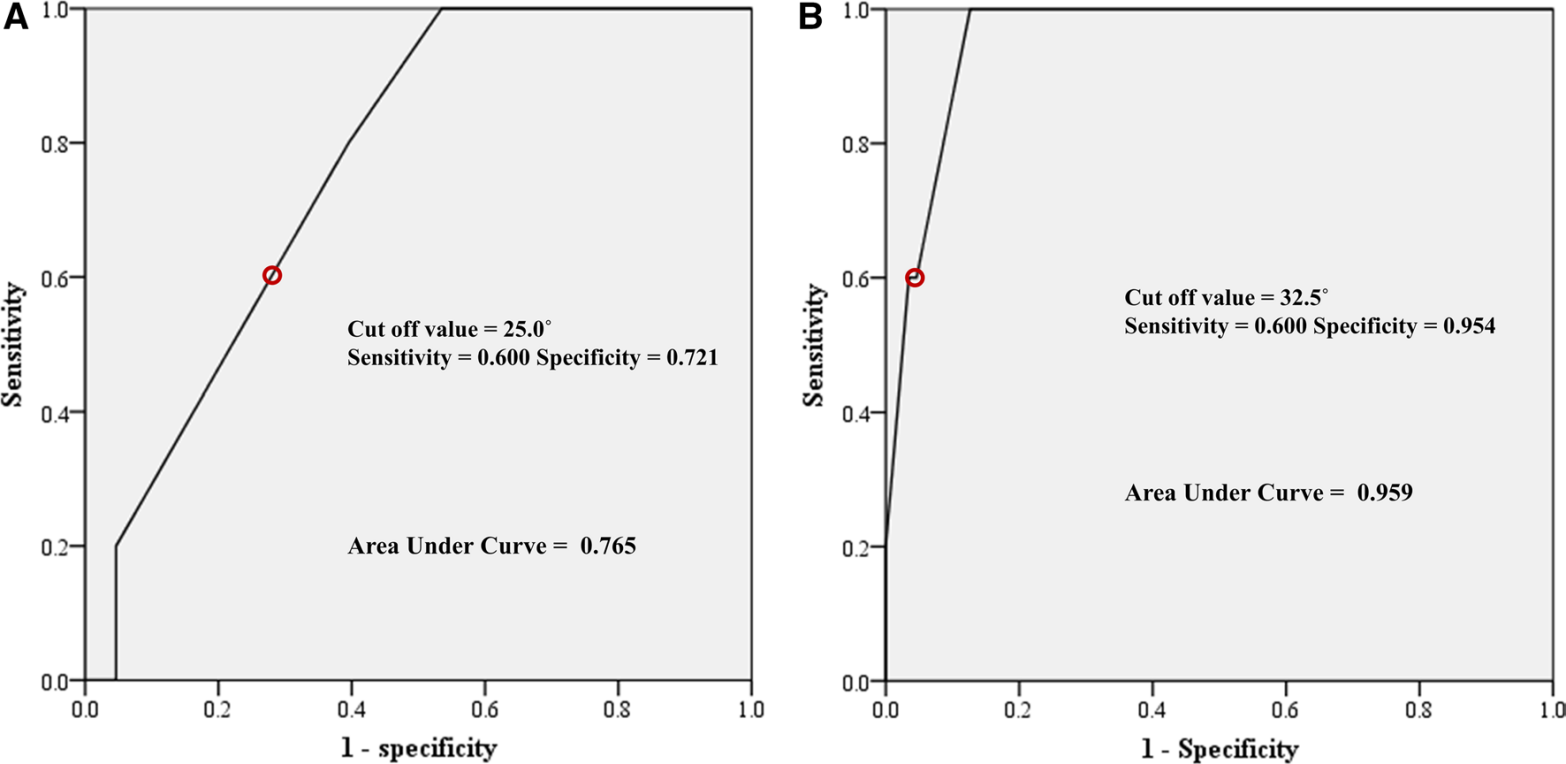
ROC curve to determine the minimal amount of preoperative flexion causing residual contracture. **A** Hemophilic arthropathy. **B** Rheumatoid arthritis

Clinical score and ROM are presented in Table 2. The degree of preoperative flexion contracture was not significantly different. The degree of flexion contracture at last follow-up was greater in the HA group (5.6° versus 1.4°, *p* < 0.001). The decreasing amount of flexion contracture did not differ between the groups. Although the maximum flexion and ROM at last follow-up were smaller in the HA group than in the RA group, the increasing amount did not differ between the groups. The maximum flexion angle at last follow-up positively correlated with the preoperative maximum flexion angle in the HA and RA groups (Fig. 1). There was a wide range of pre- and postoperative maximum flexion angle on the scattergram, and the maximum flexion angle was decreased after TKA in 17 knees (35.4%) of the HA group (Fig. 1A). The RA groups had minimal 95° of postoperative maximum flexion angle in every knee (Fig. 1B). Flexion contracture at last follow-up was positively correlated with preoperative flexion contracture in both the HA and RA groups (Fig. 2). There was a wide range of preoperative flexion contracture on the scattergram, which was decreased to < 15° in most patients after TKAs (Fig. 2A, B). However, five knees (10.4%) in the HA group and four (4.3%) in the RA group had residual flexion contracture of > 15° (*p* = 0.274). When evaluating the cutoff point of preoperative flexion contracture for postoperative residual contracture > 15° at last follow-up on ROC analysis, the cutoff value was 25.0° in the HA group and 32.5° in the RA group (Fig. 3). Significant postoperative extension lag was not observed, especially in cases with intraoperative rectus snip or V–Y quadricepsplasty.

### Radiographic evaluation

**Fig. 4:**
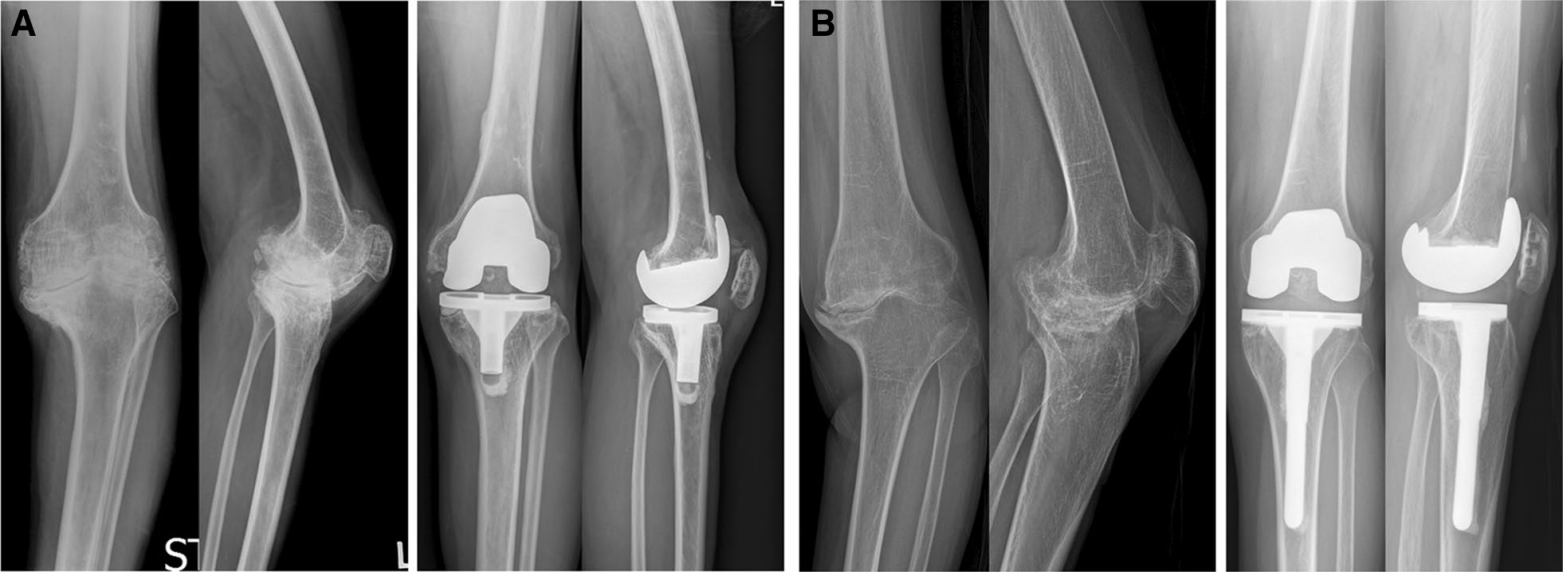
Improvement of flexion contracture after TKA in patients with severe flexion contracture of > 30°. **A** Correction of flexion contracture in hemophilic arthropathy. The preoperative flexion contracture was decreased, but sustained after TKA in a patient with hemophilic arthropathy. **B** Correction of flexion contracture in rheumatoid arthritis. The preoperative flexion contracture was fully corrected after TKA in a patient with rheumatoid arthritis

The pre- and postoperative mechanical axis (MA) results are presented in Table 3. Half (24/48 knees) of the patients in the HA group and 21.7% (20/92 knees) of those in the RA group had preoperative valgus alignment. The proportion of aligned postoperative MA within ±3° was 39 knees (81.3%) in the HA group and 84 knees (85.7%) in the RA group (*p* = 0.084). The position of components did not differ between the groups (Table 3). Fourteen knees (29.2%) in the HA group and four knees (14.3%) in the RA group had patella baja preoperatively, which remained postoperatively (Table 4; Fig. 4). Postoperative pseudo-patella baja newly occurred in five knees (10.4%) in the HA group and eight knees (8.7%) in the RA group (Table 4).

**Table 3:** Radiographic results including patella height

	Hemophilia	Rheumatoid arthritis	*p*-Value
Number of knees	48	92	
Mechanical axis (°)^a^			
Preoperative	Valgus 0.7 ± 8.6 (−18.6 to 17.4)	Varus 5.4 ± 8.9 (−23.9 to 24.9)	< 0.001
Varus knees (*n* = 24/72)	Varus 6.4 ± 4.7 (−18.6 to −1.0)	Varus 9.0 ± 5.3 (−23.9 to −0.9)	0.036
Valgus knees (*n* = 24/20)	Valgus 7.8 ± 4.9 (1.1–17.4)	Valgus 7.6 ± 6.7 (0.5–24.9)	0.913
Postoperative	Valgus 0.6 ± 2.3 (−4.2 to 5.2)	Valgus 0.3 ± 1.9 (−3.8 to 4.8)	0.467
Aligned knees with mechanical axis of ≤ ±3°	39	84	0.084
Outlier of > ±3°	9	8	
Position of components (°)			
*α* angle	95.9 ± 1.7 (93.1–100.9)	95.9 ± 1.7 (92.2–102.1)	0.990
*β* angle	89.7 ± 1.4 (86.6–92.4)	90.5 ± 1.6 (87.1–93.7)	0.206
*γ* angle	4.6 ± 2.2 (0.8–8.9)	3.2 ± 1.8 (0.5–8.4)	0.105
*δ* angle	87.7 ± 2.4 (82.4–91.8)	87.3 ± 2.1 (81.3–92.3)	0.248

^a^Negative values represent varus alignment; positive values indicate valgus alignment of the knee

**Table 4:** Incidence of patella baja and pseudo-patella baja

	Hemophilia	Rheumatoid arthritis	*p*-Value
Preoperative			
Normal/patella baja^a^/PPB^b^	34/14/0	88/4/0	< 0.001^‡^
Postoperative			
Normal/patella baja^a^/PPB^b^	28/15/5	80/4/8	< 0.001^‡^

^‡^Statistical significance was analyzed in comparison of postoperative incidence of patella baja or pseudo-patella baja in patients with normal preoperative patellar height

^a^Patella baja: both Insall–Salvati ratio (< 0.8) and Blackburne–Peel ratio (< 0.54) are abnormally low

^b^PPB (pseudo-patella baja): normal Insall–Salvati ratio (0.8–1.2) but low Blackburne–Peel ratio (< 0.54)

### Complications and additional surgeries

In the HA group, there were 19 bleeding and hemarthrosis cases (Table 5). Twelve knees were treated with increased amounts of coagulation factor concentrate, and seven were treated with incision and drainage of the hematoma. In the RA group, one intraoperative grade 2 avulsion injury of the medial collateral ligament required change of postoperative rehabilitation protocol and bracing (Table 5). Six knees in the HA group and one in the RA group had stiffness and required manipulation under anesthesia (*p* = 0.007). There was one periprosthetic fracture of the distal femur in the HA group, which was treated with open reduction and internal fixation. Three patients with PJI were treated with stage 2 revision TKA in the HA group (*p* = 0.039). There were one case of femoral component loosening in the HA group and two cases of loosening in the RA group, which required revision TKAs.

**Table 5:** Complications after primary TKA in hemophilic arthropathy and rheumatoid arthritis

	Treatment	Hemophilic arthropathy	Rheumatoid arthritis	*p*-Value
		Number of knees (%)	Number of knees (%)	
Bleeding	Extra dosing of coagulation factors	12 (25.0%)	0	< 0.001
	Incision and drainage of hematoma	7 (14.6%)	0	0.008
MCL injury	Staple fixation and change of rehabilitation	0	1 (1.1%)	> 0.999
Stiffness	Manipulation under anesthesia	6 (12.5%)	1 (1.1%)	0.007
Intraoperative fracture	Cannulated screw fixation	1 (2.1%)	3 (3.3%)	> 0.999
Postoperative periprosthetic fracture	Open reduction and fixation	1 (2.1%)	0	0.343
PJI	Two-stage revision TKA	3 (6.3%)	0	0.039
Femoral component loosening	Revision TKA	1 (2.1%)	2 (2.2%)	> 0.999
Peroneal nerve palsy	Conservative management	1 (2.1%)	0	0.343
Wound necrosis	Wound revision	0	1 (1.1%)	> 0.999
Instability	Knee brace	0	1 (1.1%)	> 0.999

*MCL* medial collateral ligament, *PJI* periprosthetic joint infection

### Survivorship analysis

**Fig. 5:**
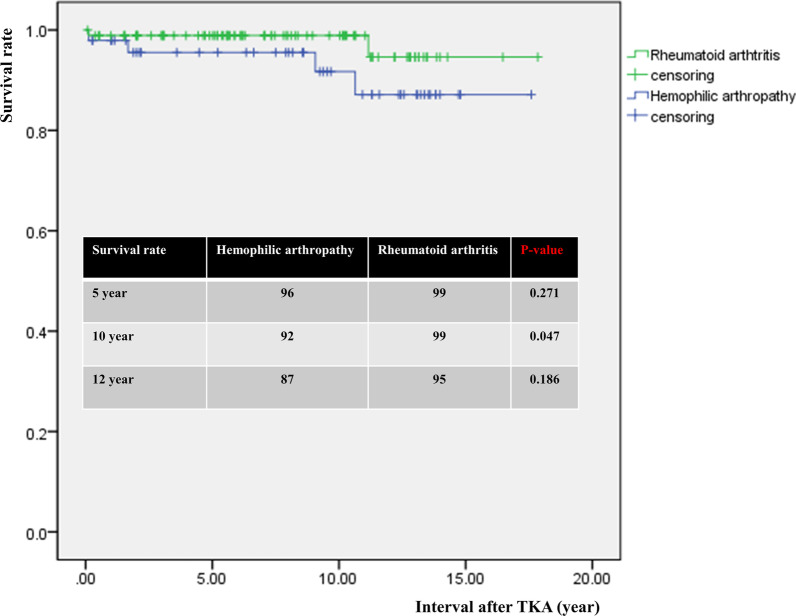
Comparison of survival rate after TKA between hemophilic arthropathy and rheumatoid arthritis

In the HA group, three knees (6.3%) underwent revision TKA at 3 months, 1.7 years, and 9.1 years after primary TKA due to PJI. One knee underwent revision TKA at 10.6 years after primary TKA due to loosening. In the RA group, two knees (2.2%) underwent revision TKA at 6 months and 11.2 years after primary TKA due to loosening. The 5- and 12-year survival rates were 96% and 87% in the HA group and 99% and 95% in the RA group, respectively (Fig. 5).

## Discussion

The most important finding of the present study is that the residual flexion contracture after TKA in end-stage HA was larger than that in the RA group, although there was no difference in the preoperative flexion contracture. In a previous study with long-term follow-up after TKA in 112 RA patients (176 knees) [19], the average flexion contracture was 27.0° preoperatively and 1.8° at last follow-up. Contrary to HA, complete correction of the preoperative flexion contracture is not obligate during TKA for RA patients because it can be gradually corrected and extension exercise is effective after TKA [13]. However, aggressive application of surgical strategies and rehabilitation protocol seems necessary for RA patients with severe preoperative flexion contracture from the data of the present study because four knees (4.3%) in the RA group had residual flexion contracture > 15°.

The second most important finding is that the cutoff value of preoperative flexion contracture to avoid a residual contracture > 15° at last follow-up was 7.5° smaller in the HA group than that in the RA group (25.0° in the HA group versus 32.5° in the RA group) (Fig. 3). These data reveal that the amount of preoperative flexion contracture should be more actively considered in HA than in RA when selecting the timing of TKA. Kubes et al. [30] reported that hemophilic patients should be operated before the preoperative flexion contracture reaches 22° to obtain a contracture < 15° postoperatively or before the preoperative contracture reaches 12° to obtain a contracture < 5°. In HA, repeated intraarticular bleeding occurs with subsequent intraarticular deposition of hemosiderin and iron, which leads to upregulation of proinflammatory cytokines and, consequently, synovial hypertrophy and articular destruction [31]. Therefore, it is thought that attentively passive and assisted active exercise of knee flexion–extension is necessary to decrease the risk of periarticular bleeding with proper factor replacement during rehabilitation after TKA in HA. Contrary to in HA, RA patients experience less intracapsular fibrotic changes and extracapsular muscle contractures and seem to have gradual correction of flexion contracture after TKA.

Another important finding of this study is that postoperative hemarthrosis was one of the common complications in HA, with prevalence rates of 25% and 8.9% in the present study and the previous metaanalysis, respectively [32]. Wong et al. [21] recommended coagulation factor replacement at a higher level for a longer period than that in the current World Hemophilia Foundation guidelines. We believe that the prevalence of bleeding might have been decreased if we had used a high coagulation factor replacement regime, maintaining a 100% level of coagulation factor throughout the first 3 weeks following TKA [33]. Most studies about TKA in HA and RA indicate that complications such as PJI and loosening are more frequent than in patients with degenerative osteoarthritis [3, 15–18]. With a special emphasis on late PJI, Rodriguez-Merchan et al. [34] reported an infection rate of 6.8% (early infection 1.1%, late infection 5.7%) in TKA in HA. The cause of the high incidence of PJI after TKA in HA is still unknown [7], but it may be due to poor skin condition caused by coagulation factor administration, immunosuppression, concomitant human immunodeficiency virus (HIV) or hepatitis C virus (HCV) infection, or bacteremia from contamination during repeated intravenous self-administration of coagulation factor concentrate [1, 33]. In the HA groups of the present study, three knees (6.3%) underwent revision TKA at 3 months, 1.7 years, and 9.1 years after primary TKA due to PJI. The low incidence of late PJI in our cohort may have been because the patients were well supervised by KHF with education on meticulous sterility during self-infusion, and regular medical checkups in addition to the low proportion of immunosuppressed patients positive for HIV (Table 1).

Solimeno et al. [35] reported an aseptic loosening rate requiring revision TKA of 6% in HA patients with a median time to implant removal of 7 years. In the present study with mean follow-up of 8.8 years, there was one loosening at 10.6 years after TKA for HA patients. Young age, poor bone quality, microhemorrhages, and reactive destructive reactions at the bone–cement interface are potential causes of the high incidence of aseptic loosening [7]. HA patients are generally younger than those with osteoarthritis and are expected to have higher levels of physical activity after TKA, so they may have a higher risk of loosening. However, Zingg et al. [36] noted that HA patients generally have few demands in daily living and have adapted to their musculoskeletal problems since childhood. Interestingly, there were two incidences of loosening at 6 months and 11.2 years after TKA in RA patients in the present study with mean follow-up of 8.0 years. HA and RA patients had similar incidence of loosening after TKA and period of revision TKA. This finding shows that the period of loosening should be considered in selecting the timing of TKA for HA and RA.

The present study has several limitations. The principal limitation is its retrospective nature with small sample size. However, it would be difficult to conduct a prospective study or a matched pair analysis when considering the rare incidence of TKA in HA and RA patients. Second, differences existed in demographics such as age, sex, body mass index, and duration of disease between the HA and RA groups. But considering the etiology and pathophysiological characteristics of HA and RA, these differences were inevitable. Third, serial change of flexion contracture after TKA was not investigated in our study. This would be an interesting issue because flexion contracture could get worse with time in the HA group (due to recurrent hemarthrosis and subsequent fibrosis), but not in the RA group. Further study on this issue will be required. Last, TKA procedures were performed by experienced surgeons (S.J.S. and D.K.B.) in a tertiary medical center with a good hemophilia care system. This means that caution must be taken when seeking to extrapolate our findings to countries with different medical situations and general hospitals.

Despite some limitations, data from the present study can be used to inform HA and RA patients about the threshold of preoperative flexion contracture for the risk of residual flexion contracture, complications, and survival rate after TKA.

## Conclusion

The postoperative residual flexion contracture was greater and the cutoff value of preoperative flexion contracture for residual contracture was smaller in the HA group than the RA group. Appropriate intra- and postoperative care to avoid postoperative residual contracture is required in HA patients.

## Data Availability

Not applicable.

## Abbreviations

TKA: Total knee arthroplasty
HA: Hemophilic arthropathy
RA: Rheumatoid arthritis
ROM: Range of motion
OA: Osteoarthritis
PJI: Periprosthetic joint infection
KHF: Korea Hemophilia Foundation
WOMAC: Western Ontario and McMaster Universities Arthritis Index
PACS: Picture archiving and communication system
ISR: Insall–Salvati ratio
BPR: Blackburne–Peel ratio
ROC: Receiver operating characteristics
MA: Mechanical axis
HIV: Human immunodeficiency virus
HCV: Hepatitis C virus
